# Giant primary pleomorphic adenoma of the lung presenting as a post-traumatic pulmonary hematoma: a case report

**DOI:** 10.1186/s13019-016-0409-z

**Published:** 2016-01-21

**Authors:** Zrinka Požgain, Grgur Dulić, Jozo Kristek, Jasmina Rajc, Siniša Bogović, Marko Rimac, Izabela Kiš

**Affiliations:** Department of Thoracic Surgery, Surgery Clinic, Osijek University Hospital, Josipa Huttlera 4, HR-31000 Osijek, Croatia; Department of Cardiac Surgery, Surgery Clinic, Osijek University Hospital, Josipa Huttlera 4, HR-31000 Osijek, Croatia; Pathology Clinic, Osijek University Hospital, Josipa Huttlera 4, HR-31000 Osijek, Croatia; Department of Pediatric Surgery, Surgery Clinic, Osijek University Hospital, Josipa Huttlera 4, HR-31000 Osijek, Croatia; Faculty of Medicine, J.J. Strossmayer University of Osijek, Cara Hadrijana 10E, HR-31000 Osijek, Croatia

**Keywords:** Adenoma, Pleomorphic, Hematoma, Thoracic injuries

## Abstract

**Background:**

Pleomorphic adenomas, also known as benign mixed tumors, are the most common tumors of glandular origin in the head and neck and although they are generally benign they can undergo malignant transformation. Primary pleomorphic adenomas of the lung are extremely rare tumors with less than 40 cases reported in the literature by now. This is the first case in the literature describing overlapping with a traumatic event and also one of the rare cases describing primary adenoma of the lung reaching this impressive size.

**Case presentation:**

We report a rare case of a giant primary pleomorphic adenoma of the lung presenting as a post-traumatic pulmonary hematoma. A 38-year-old Caucasian male patient came to the Urgent Trauma Center after being hit in the chest by a bull and, after a number of tests, was diagnosed with primary pleomorphic adenoma of the lung. Operative treatment was performed and the surgical excision was successfully done.

**Conclusions:**

Our conclusion is that the surgical excision is the main treatment for pleomorphic adenoma of the lungs and we recommend lifelong follow-up and regular check-ups. Furthermore, we consider our case an interesting one due to its concurrence with the chest trauma and the dilemma about the optimal approach considering the entity could have been a large interlobular hematoma.

## Background

Pleomorphic adenomas, also known as benign mixed tumors, are the most common tumors of glandular origin in the head and neck and although they are generally benign they can undergo malignant transformation. Metastasizing mixed tumors have been reported to metastasize to bones, lymph nodes and lungs [1]. Primary pleomorphic adenomas of the lung are extremely rare tumors with less than 40 cases reported in the literature at present. Primary pulmonary pleomorphic adenomas have predominately occurred in the proximal airway with only a few reported cases of primary pleomorphic adenoma in the lung periphery [2]. We report a rare case of a giant primary pleomorphic adenoma of the lung presenting as a post-traumatic pulmonary hematoma.

**Fig. 1:**
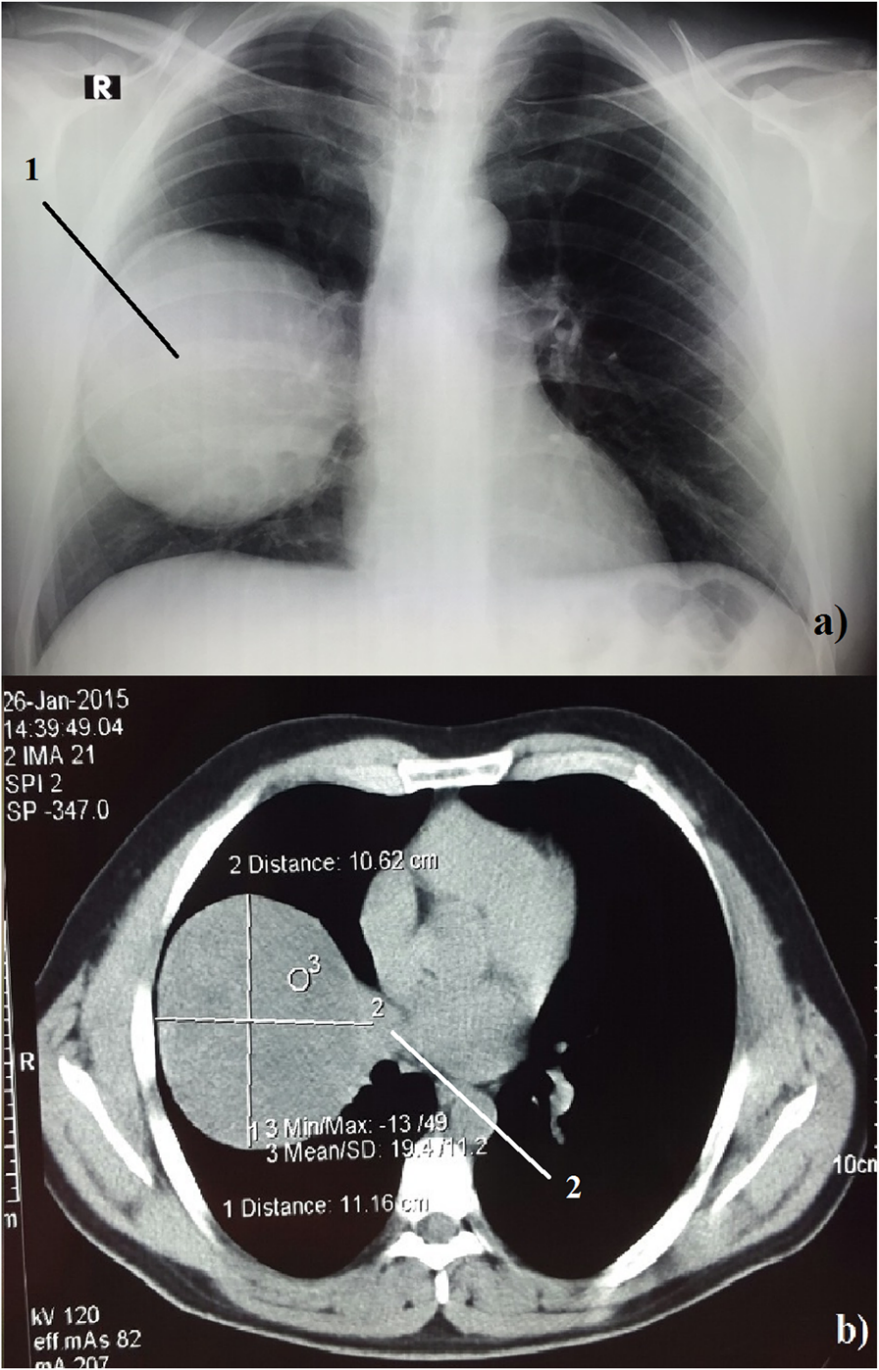
**a** Patient’s pulmonary X-ray image with a large tumor located in the right lung (10,6x11,1x11,6 cm) (1). **b** CT-scan image of the patient’s lungs. The tumor is originating from the right main bronchus (2)

**Fig. 2:**
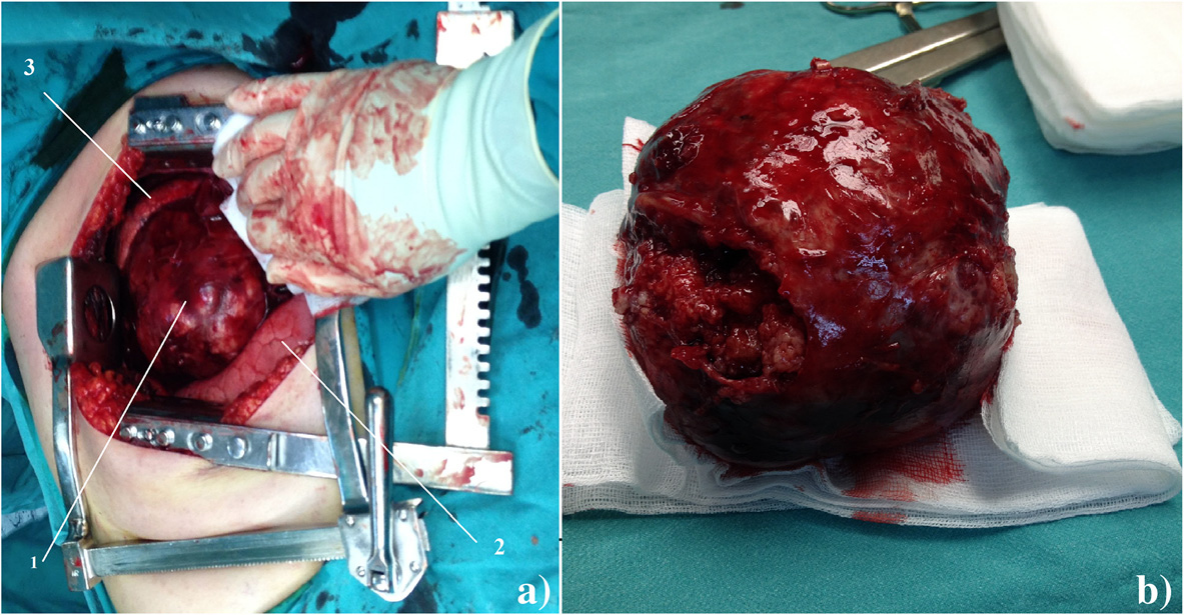
**a** Pleomorphic adenoma in the right lung. 1 – Tumor, 2 – Lower lobe of the right lung, 3 – Upper lobe of the right lung. **b** Pleomorphic adenoma after the extirpation

**Fig. 3:**
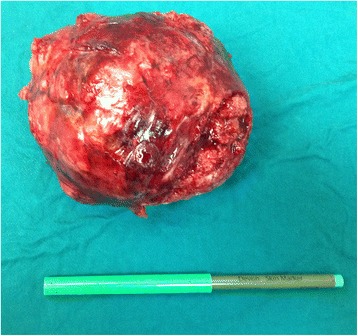
Pleomorphic adenoma after the extirpation next to a ruler

## Case presentation

A 38-year-old male patient came to the Urgent Trauma Center after being hit in the chest by a bull. Symptoms were mild pain on the right lower ribs posteriorly without visible contusion marks and without cardiorespiratory compromisation. According to the patient’s medical history he had never had any respiratory symptoms or condition before the incident. A chest X-ray was performed and while there was no sign of broken ribs, pleural effusion or pneumothorax, there was a big, sharply marked, round shadow present on the right lung. Laboratory tests (complete blood count, C-reactive protein, renal function and coagulation parameters), thorax and abdominal ultrasound were performed immediately without any pathological finding. Urgent thorax computed tomography (CT) scan was performed and the radiologist described a dense sharply marked formation filled with fluid sized 10.6 × 11.1 × 11.6 cm in contact with the right main bronchus (Fig. 1). The patient was admitted, a bronchoscopy was performed and there was no intraluminal evidence of tumor. A pulmonologist was called in, who then recommended an ultrasound or a CT-guided thoracic puncture considering the formation to be an intrapulmonary hematoma and, less likely, a primary lung tumor or an echinococcal cyst. CT-guided transthoracic puncture was performed and the substance was sent for a cytological analysis which showed that the formation is most likely a pleomorphic adenoma but could also be a hamartoma and required histopatholological verification. From the admittance and during his hospital stay the patient was stable and asymptomatic. The operation procedure was scheduled as soon as the cytological analysis was completed. Right lateral muscle-sparing thoracotomy was performed. Macroscopic examination showed a large reddish capsulated tumor with a smooth surface, clearly demarked from the surrounding lung parenchyma. There were no macroscopic pathological findings on the pulmonary lobes. During the extirpation, surgical resection of the tumor from the right main bronchus was done using the ultracision. The tumor was extirpated and we noticed that the capsule on the extirpated tumor was not intact. A small portion of the tumor was still on the right main bronchus. The resection of the remaining tumor was performed, after which the right main bronchus seemed to have macroscopically clear resection margin (Fig. 2). Hilar station lymphadenectomy was performed and the nodes were sent with a tumor for an intraoperative histopathological analysis showing no malignancy. Postoperative recovery was uneventful. Results of the final histopathological analysis verified it was truly a pleomorphic adenoma. Considering this result, the patient was sent to do a salivary gland CT-scan which did not show any pathological finding. After the operation a positron emission tomography (PET) CT was performed, which did not show any local or distant metastasis. Since the PET CT did not show any bone metastasis we did not consider a bone scan necessary.

## Discussion

Pleomorphic adenoma is the most common tumor of the major salivary glands, usually developing in the palate, tongue, nasopharynx or larynx. Its primary location in the lungs is extremely rare and it usually arises from the tracheal and bronchial seromucous glands [2, 3]. Pulmonary adenomas usually include bronchial adenoma, alveolar adenoma, papillary adenoma and adenomas of the salivary gland while primary salivary-type lung cancers are rare tumors that include adenoid cystic carcinoma and mucoepidermoid carcinoma. Pleomorphic adenoma constitutes about 1 % of all the cases of primary lung adenomas [2]. Considering age and gender there has not been any proven gender predominance and the patients’ age ranges from 8 to 74 [3], but it seems that they occur more common in younger patients. Clinically, pleomorphic adenoma of the lungs may present with dyspnea and hemoptysis or fever, weight loss, pleural effusion and shortness of breath depending on its location or frequently lacking any symptoms [4], as it occurred in our case. The tumor in our case originated from the right main bronchus according to the thorax CT-scan, which is in lieu with the fact that the pleomorphic adenomas of the lung are commonly centrally positioned. The size of the tumor was 10.6 × 11.1 × 11.6 cm making it one of the biggest pleomorphic adenomas of the lungs considering the literature stating that the size varies from 1.5 to 16 cm in diameter [5] (Fig. 3). Differential diagnosis of primary finding on the X-ray and CT-scan included large interlobular hematoma, echinococcal cyst, hamartoma, and other pulmonary tumors such as blastoma, hamartochondroma and carcinosarcoma. We decided to perform an early surgical procedure because cytological results did not offer a certain response about the nature of the formation. Also, an early surgical procedure was opted for due to the large size and the possibility of a hematoma. Other invasive diagnostic procedures such as endobronchial ultrasound (EBUS) were considered but were not performed after the decision was made about the early surgical procedure. Subsequently, a PET CT was considered preoperatively due to the possibility of the metastasis but since the metastases were described in very few cases it was done postoperatively. The surgical excision is the main treatment for pleomorphic adenoma of the lungs [5]. Although in our case the tumor originated from the right main bronchus, since the pathologist intraoperatively stated that there were no malignant cells present, a sleeve resection and a bronchoplasty were not performed. We decided to wait for the final histopathological diagnosis. If the diagnosis contained findings of malignant cells or unclear resection margins, a more extensive procedure would have been performed in subsequent treatment. Considering the intraoperative histopathology, hilar station lymphadenectomy was deemed sufficient in our case. If the final histopathological diagnosis had indicated so, then we would have performed further mediastinal and aortic lymphadenectomy using video-assisted thoracoscopic surgery (VATS) [6]. The final histopathological diagnosis confirmed it was a pleomorphic adenoma consisting of epithelial elements and tubules with uniform cells and well-shaped round nuclei. Between these cells there was a lot of myxoid stromal and partially chondroid tissue. Considering the size of the tumor in our case video-assisted thoracoscopic surgery (VATS) was not performed, but in cases with smaller tumors VATS is recommended. There have been reported cases of recurrence after surgical excision wherein the metastases where present locally by pleural dissemination or there were distant metastases in the breast and spine [5]. This recurrence may be caused by incomplete excision or due to a core needle biopsy [5, 7].

## Conclusions

In literature there are various follow-up strategies, ranging from 9 months to 18 years [8]. We therefore recommend lifelong follow-up and regular check-ups, including a thorax CT scan every 6 months during the first year, followed by a thorax CT scan once a year [5, 7, 8]. Another reason why we find our case interesting is the coincidence with the chest trauma and the dilemma about an optimal approach considering the object could have been a large interlobular hematoma. Considering the lack of symptoms, we recommend this object be included in the differential diagnosis in patients with abnormalities on the pulmonary X-ray.

## Consent

Written informed consent was obtained from the patient for publication of this Case report and any accompanying images. A copy of the written consent is available for review by the Editor of this journal.
